# Seed Hormonal Priming Improves Drought Resilience in Durum Wheat Through Modulation of Physiological and Biochemical Traits

**DOI:** 10.3390/plants15111700

**Published:** 2026-05-30

**Authors:** Rihab Zagoub, Manel Hmissi, Erika Fernandez-Martinez, Francisco Garcia-Sanchez, Abdelmajid Krouma

**Affiliations:** 1 Laboratory of Ecosystems and Biodiversity in Arid Land of Tunisia, Faculty of Sciences, University of Sfax, Sfax 3029, Tunisia; zagoubrihab123@gmail.com (R.Z.); hmissimanel567@gmail.com (M.H.); abdelmajid.krouma@fstsbz.rnu.tn (A.K.); 2 Faculty of Sciences and Techniques of Sidi Bouzid, University of Kairouan, Kairouan 3100, Tunisia; 3 Centro de Edafología y Biología Aplicada del Segura (CEBAS-CSIC), E-30100 Murcia, Spain; efernandez@cebas.csic.es

**Keywords:** antioxidant enzymes, drought, durum wheat, hormonal priming, osmolytes, water relations

## Abstract

Drought stress is one of the most severe constraints affecting wheat production worldwide. Under these conditions, the development of sustainable and economically viable strategies, such as seed priming, is essential to improve wheat performance and drought resilience. The present study carried out a greenhouse experiment on four Mediterranean durum wheat cultivars (*Triticum turgidum* ssp. *durum* Desf), i.e., Karim (Kr) and Khiar (Kh) from Tunisia and Espelta (Esp) and Mocho (Mo) from Spain, subjected to drought stress conditions, and using primed abscisic acid (ABA), indole-3-acetic acid (IAA), melatonin (Mlt), and salicylic acid (SA), and non-primed seeds. In order to assess the physio-biochemical responses of durum wheat, such as plant growth, chlorophyll, relative water content (RWC), water potential (Ψw), osmotic potential (Ψs), proline, soluble sugars, starch, glycine betaine, hydrogen peroxide, malondialdehyde, and antioxidant enzyme activities. The results showed that water stress significantly reduced plant growth, SPAD index, RWC, Ψw, and Ψs, while upregulating H_2_O_2_ and MDA levels, depending on the wheat cultivars. Soluble sugars decreased, whereas starch, glycine betaine, and proline accumulated in all cultivars. Superoxide dismutase activity was reduced (24–37%) under water stress as compared to the control condition, while APX, CAT, and POD activities significantly increased. Among the cultivars, Esp exhibited the greatest plasticity in response to water deficit, whereas Kh appeared to be most sensitive. Furthermore, the present results revealed that the priming durum wheat seeds with ABA, IAA, Mlt, and SA improved leaf hydration, particularly through soluble sugar accumulation. Seed priming also alleviated oxidative stress by reducing H_2_O_2_ and MDA levels and stimulating APX, CAT, POD, and SOD activities. Plants grown from non-primed seeds of Spanish and Tunisian cultivars exhibited differential responses to drought stress, and those derived from primed seeds showed varying degrees of enhanced drought tolerance. Espelta demonstrated a high potential for stress tolerance and responsiveness to priming, followed by Karim, whereas Khiar was the most sensitive cultivar. Overall, the cultivars can be ranked in decreasing order of stress tolerance as Esp > Kr > Mo > Kh. These findings highlight the potential of phytohormone-based seed priming as an efficient and practical approach to enhance drought resilience in durum wheat, offering promising prospects for improving crop performance and stability under increasingly water-limited conditions in the era of climate change.

## 1. Introduction

Water scarcity constitutes one of the most critical constraints to agricultural production worldwide. According to Toretti et al. [1], droughts not only disrupt ecosystems but also inflict severe agricultural losses, thereby undermining food security and rural livelihoods. Drought is commonly defined as a prolonged deficiency in water availability, manifesting in several forms: meteorological drought (below-average rainfall), agricultural drought (insufficient irrigation supply), hydrological drought (reduced surface and groundwater reserves), and physiological drought (when plant transpiration exceeds water uptake) [2]. Among all sectors, agriculture is the most vulnerable to water scarcity, with yield potential in rain-fed regions declining substantially by 40–60% [3].

Drought stress is broadly categorized into different stages: escape, avoidance, and tolerance [4,5]. Physiologically, drought induces stomatal closure mediated by abscisic acid (ABA), which reduces carbon assimilation and alters hormonal signaling pathways, while also weakening root development and nutrient uptake capacity [6]. Morphological adaptations include enhanced root growth, reduced stomatal density, decreased leaf area, increased leaf thickness, biosynthesis of cuticular waxes on shoots, and leaf folding, all of which contribute to minimizing evapotranspiration and conserving water [7,8]. Drought stress also impairs photosynthetic efficiency, restricts nutrient acquisition, and accelerates oxidative damage, ultimately leading to reduced biomass accumulation and yield [9]. These disruptions are further compounded by oxidative stress, where reactive oxygen species (ROS) accumulate and damage cellular structures, thereby diminishing plant resilience [10]. Beyond the biological scale, recurrent droughts exacerbate socio-economic vulnerabilities by reducing farm revenues, destabilizing rural communities, and increasing dependence on external food imports.

Durum wheat (*Triticum turgidum* ssp. *durum* Desf.) is a vital cereal crop and one of the most widely consumed staple foods worldwide, providing a major source of carbohydrates, proteins, and calories in the human diet [11]. To satisfy the nutritional requirements of a global population projected to reach 9.7 billion by 2050, wheat production must increase by approximately 70% compared to current levels [12]. Terminal drought episodes occurring during the grain-filling stage severely impair photosynthesis, reduce chlorophyll content and carbon assimilation, and ultimately result in yield losses and deterioration of grain quality traits, including protein content and gluten strength [13]. Nevertheless, durum wheat genotypes exhibit substantial genetic variability, with landraces and traditional varieties frequently demonstrating adaptive traits such as osmotic adjustment, deeper rooting systems, and enhanced antioxidant activity, which collectively confer improved tolerance to water deficit [14].

Among the various approaches used to alleviate abiotic stress, seed priming has emerged as an efficient method that improves seedling emergence and growth, shortens the crop cycle, and ultimately enhances yield potential [15]. A range of priming techniques has been developed to improve crop performance under stress, including hormonal priming, osmopriming, hydropriming, stratification, humidification, hardening, and thermal shock [16]. Among these, hormonal priming is considered a particularly effective and economical strategy; however, its success largely depends on factors such as plant species, seed viability, the concentration of the priming agent, and the protocol employed [16]. The positive effects of priming are mainly linked to its capacity to maintain seed integrity, especially by protecting cellular membranes during imbibition, while also promoting DNA repair, the synthesis of RNA and proteins, and antioxidative defense mechanisms [17]. Furthermore, exogenous seed priming with signaling molecules such as melatonin, salicylic acid, abscisic acid, and auxin has been shown to significantly enhance plant tolerance to drought stress by improving antioxidant activity, osmotic adjustment, stomatal regulation, and root development, thereby sustaining growth and productivity under adverse conditions [18].

Melatonin (Mlt), due to its multifunctional properties, plays a significant role in enhancing plant tolerance to drought and other abiotic stresses. It acts by scavenging reactive oxygen species (ROS), stimulating photosynthesis, and regulating numerous metabolic pathways. Melatonin is synthesized in chloroplasts and mitochondria [19,20], and its endogenous levels vary depending on environmental conditions. As a protective compound, it accumulates in plants exposed to various stresses, such as water deficit, flooding, extreme temperatures, ultraviolet (UV) radiation, and heavy metal toxicity [21]. In addition to its antioxidant activity, melatonin modulates the expression of stress-related genes, thereby improving photosynthetic performance and facilitating water uptake under limited water availability [21,22]. Furthermore, its use as a priming agent has been shown to enhance lateral root development and accelerate seed germination under water stress conditions [23].

Seed priming with SA has been shown to enhance drought resilience by activating antioxidant enzymes, maintaining chlorophyll stability, and promoting osmotic adjustment, thereby sustaining metabolic activity under water deficit conditions [24,25]. In addition, SA priming increases the accumulation of osmolytes such as proline and glycine betaine, which play a crucial role in mitigating the adverse effects of abiotic stresses [26,27]. Furthermore, rice seeds primed with SA at 100 ppm have been reported to exhibit improved germination and accelerated seedling growth under polyethylene glycol (PEG)-induced water stress [28].

Abscisic acid (ABA) is critically involved in mediating adaptive mechanisms under drought and high temperature, thereby contributing to plant survival and resilience [29,30]. ABA is primarily synthesized in plastids of leaves and roots, with its production markedly enhanced under drought stress [31]. During water deficit, ABA plays a pivotal role in drought adaptation by inducing stomatal closure to reduce transpiration, stimulating the accumulation of osmolytes, and modulating root architecture to improve water-use efficiency [32]. Jakab et al. [33] further demonstrated that priming *Arabidopsis* seeds with amino-butyric acid enhanced drought tolerance by promoting ABA accumulation and inducing stomatal closure.

Auxins, such as indole-3-acetic acid (IAA), play a fundamental role in regulating plant growth and developmental processes, including root initiation and elongation, cell expansion, vascular tissue differentiation, and the maintenance of apical dominance [34]. Auxins are synthesized predominantly in the shoot apex and young leaves and are transported basipetally toward the roots via the polar auxin transport system [35,36,37]. Although primarily associated with growth regulation, auxins also contribute to drought tolerance by stimulating root elongation and lateral root formation, thereby increasing the plant’s capacity to absorb water and nutrients under stress conditions [34]. Priming seeds with auxin (IAA) at 50 ppm has been reported to improve drought tolerance by enhancing the activities of antioxidant enzymes, notably catalase (CAT), superoxide dismutase (SOD), and peroxidase (POD) [38].

The Mediterranean region is a “hotspot” for climate change, warming 20% faster than the global average (+1.3 °C at the sea surface between 1982 and 2019) [39]. This area is facing rising temperatures, droughts, wildfires, sea level rise, and intense rainfall, threatening biodiversity and 290 million people by 2050 [40]. The coastal regions of the north and south Mediterranean are currently facing critical challenges, including water scarcity, food and energy insecurity, and ecosystem degradation, including agroecosystems. In this context of ongoing climate change, identifying strategies to enhance crop resilience to water scarcity has become a critical priority for sustainable agriculture and food security, particularly in Mediterranean regions. The rich genetic diversity of durum wheat in this area represents a valuable resource for selecting genotypes with improved adaptive capacity to drought stress. Among the available approaches, seed priming with eco-friendly hormonal agents offers a promising, low-cost strategy to enhance plant tolerance and performance under adverse conditions. Within this framework, the present study aims to (i) investigate genotypic variability in the physiological and biochemical responses of durum wheat to drought stress, and (ii) evaluate the effectiveness of hormonal seed priming in improving stress resilience. To achieve this, a functional analysis was conducted on four Mediterranean durum wheat cultivars—Karim (Kr) and Khiar (Kh) from Tunisia and Espelta (Esp) and Mocho (Mo) from Spain—using seeds either primed with different hormonal agents or left unprimed, under controlled drought stress conditions.

## 2. Results

### 2.1. Plant Growth and Chlorophyll Biosynthesis

**Figure 1:**
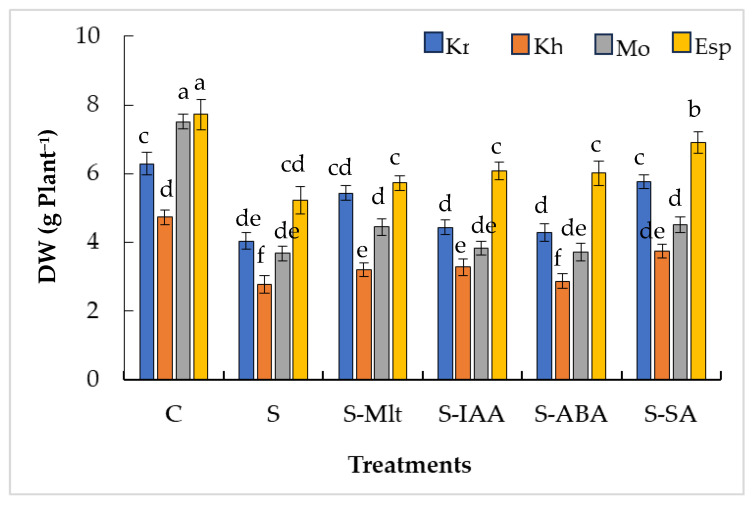
Dry biomass production of durum wheat cultivars (Kr: Karim, Kh: Khiar, Mo: Mocho, Esp: Espelta) subjected to drought stress (S) or maintained under control conditions (C), and plants exposed to drought stress following seed priming with melatonin (S-Mlt), indole-3-acetic acid (S-IAA), abscisic acid (S-ABA), or salicylic acid (S-SA). Different lowercase letters above the bars indicate statistically significant differences at *p* < 0.05 according to Fisher’s Least Significant Difference (LSD) test. Error bars represent the standard error of the mean (*n* = 10 plants).

Quantitative analysis of dry biomass production in durum wheat cultivars revealed a significant negative impact of drought stress on shoot growth. Compared with control plants, drought conditions reduced shoot biomass by 36%, 41%, 51%, and 32% in Kr, Kh, Mo, and Esp, respectively (*p* < 0.05; Figure 1). Under these stress conditions, the cultivar Esp exhibited higher dry biomass accumulation, producing 1.3, 1.9, and 1.4 times more biomass than Kr, Kh, and Mo, respectively. Nevertheless, seed hormonal priming partially mitigated the adverse effects of drought, with responses varying according to both the cultivar and the priming agent used. When primed with Mlt, shoot growth decreased by 14%, 33%, 41%, and 26% in Kr, Kh, Mo, and Esp, respectively (compared to control plants), allowing recovery of 22%, 8%, 10%, and 6% of the dry weight (DW) lost under drought stress (Figure 1). The use of IAA as a priming agent also relatively alleviated the inhibitory effect of drought stress on plant growth. Indeed, in IAA-primed plants, dry weight decreased by 29% (instead of 36% under stress compared to control), 31% (instead of 41%), 49% (instead of 51%), and 21% (instead of 32%) in Kr, Kh, Mo, and Esp, respectively. Abscisic acid also demonstrated a beneficial effect in alleviating drought stress on plant growth, with a response comparable to that of IAA. Plant biomass decreased by 32%, 51%, 35%, and 22% in Kr, Kh, Mo, and Esp, respectively (compared to control plants; Figure 1). The most pronounced beneficial effect of seed priming was observed in SA-primed plants, where biomass reductions were 32%, 51%, 35%, and 22% in Kr, Kh, Mo, and Esp, respectively, compared to control plants (Figure 1). Comparative analysis of cultivars revealed a clear superiority of Esp and, to a lesser extent, Kr over the remaining cultivars, irrespective of the treatment.

**Figure 2:**
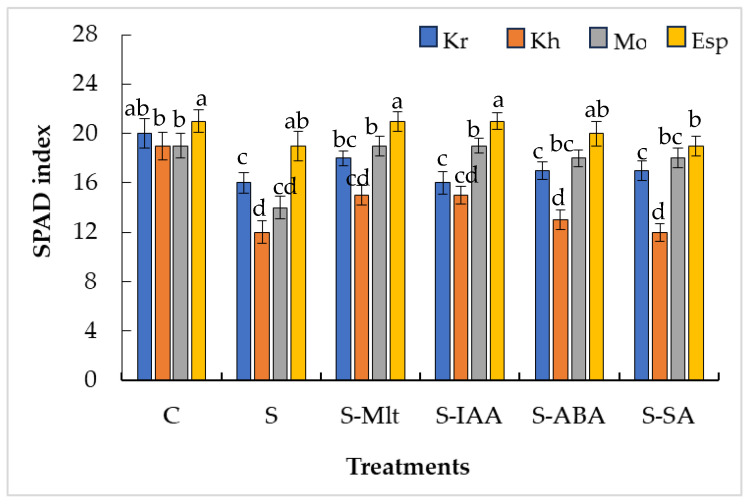
SPAD index of durum wheat cultivars (Kr: Karim, Kh: Khiar, Mo: Mocho, Esp: Espelta) grown under control conditions (C), subjected to drought stress (S), or exposed to drought stress following seed priming with melatonin (S-Mlt), indole-3-acetic acid (S-IAA), abscisic acid (S-ABA), or salicylic acid (S-SA). Different lowercase letters above the bars indicate statistically significant differences at *p* < 0.05 according to Fisher’s Least Significant Difference (LSD) test. Error bars represent the standard error of the mean (*n* = 10 plants).

The Soil–Plant Analysis Development (SPAD) index is an in situ measure that reflects leaf chlorophyll content. The SPAD index decreased significantly under drought stress (*p* < 0.05), with reductions ranging from 10% in Esp to 37% in Kh (20% in Kr and 26% in Mo; Figure 2). Seed hormonal priming effectively alleviated the detrimental effects of drought on the SPAD index, with responses varying according to cultivar and hormone type. SPAD recovery ranged from 10% to 26%, with the cultivar Esp showing the strongest response to priming, achieving full recovery of its SPAD values under certain treatments.

Chlorophyll pigments reflect shoot chlorophyll content as influenced by the different treatments. Table 1 presents the chlorophyll content in the shoots of durum wheat cultivars under the various experimental conditions. Examination of the data reveals a slight reduction in chlorophyll *a* (Chl-*a*) in Kh and Esp (−7% and −8%, respectively), and a more pronounced decrease in Kr and Mo (−12% and −15%, respectively) under drought stress. Chlorophyll *b* (Chl-*b*) exhibited a similar pattern. Hormonal seed priming mitigated the negative effects of drought on both Chl-*a* and Chl-*b*, in some cases fully offsetting the reductions. The most pronounced impact of drought stress was observed in carotenoids, which decreased by 23%, 24%, 27%, and 21% in Kr, Kh, Mo, and Esp, respectively, compared to control plants. Seed hormonal priming promoted the recovery of carotenoid biosynthesis, in some treatments even exceeding the levels observed in control plants.

### 2.2. Water Relations and Osmolytes Accumulation

Relative water content (RWC) in shoots decreased significantly under drought stress in all cultivars (*p* < 0.05; Table 2). Shoot hydration declined by 33%, 37%, 30%, and 28% in Kr, Kh, Mo, and Esp, respectively, compared to control plants. Seed hormonal priming mitigated these adverse effects, with responses varying according to cultivar and priming agent. In Kr, rehydration ranged from 18% in S-Mlt and S-ABA treatments to 24% in S-SA (20% in S-IAA). In Kh, rehydration varied from 18% to 24%, with the highest values observed under S-SA. In Mo, recovery ranged from 13% to 17%, while in Esp it ranged from 14% to 23%. Overall, salicylic acid (SA) appeared to be the most effective priming agent, inducing the greatest improvement in shoot hydration (Table 2).

**Figure 3:**
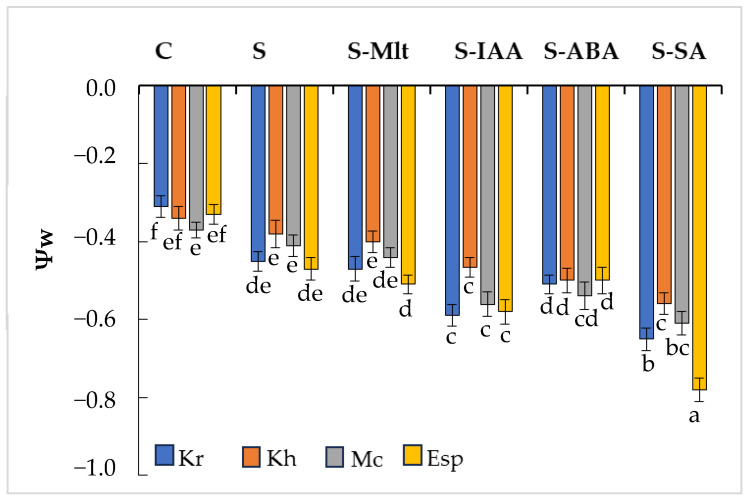
Water potential (Ψw) in durum wheat cultivars (Kr: Karim, Kh: Khiar, Mo: Mocho, Esp: Espelta) grown under control conditions (C), subjected to drought stress (S), or exposed to drought stress following seed priming with melatonin (S-Mlt), indole-3-acetic acid (S-IAA), abscisic acid (S-ABA), or salicylic acid (S-SA). Different lowercase letters above the bars indicate statistically significant differences at *p* < 0.05 according to Fisher’s Least Significant Difference (LSD) test. Error bars represent the standard error of the mean (*n* = 10 plants).

Water potential (Ψw) is an important indicator of plant water status. Drought stress significantly reduced Ψw in Kr and Esp by 45% and 42%, respectively (*p* < 0.05; Figure 3). Seed hormonal priming modulated this response depending on the applied agent. Melatonin promotes Ψw by 7%, 6%, 8%, and 12% in Kr, Kh, Mo, and Esp, respectively, compared to drought-stressed, non-primed plants (which showed reductions of 52%, 18%, 19%, and 55% in Kr, Kh, Mo, and Esp compared to control plants; Figure 3). Indole-3-acetic acid (IAA) appeared more effective, promoting Ψw by 45%, 26%, 41%, and 33% in Kr, Kh, Mo, and Esp, respectively, relative to drought-stressed, non-primed plants (which exhibited decreases of 90%, 37%, 52%, and 76% compared to controls; Figure 3). Abscisic acid (ABA) acted similarly to melatonin and IAA, with reductions in Ψw of 67%, 47%, 46%, and 52% in Kr, Kh, Mo, and Esp compared to control plants (Figure 3). Salicylic acid (SA) significantly decreased Ψw, increasing its negativity by 65%, 53%, 54%, and 94% in Kr, Kh, Mo, and Esp, respectively, compared to drought-stressed, non-primed plants (which showed decreases of 110%, 65%, and 136% in Kr, Kh, and Esp compared to controls; Figure 3). Comparison among cultivars revealed that Esp responded most favorably to seed priming, while SA demonstrated the highest efficiency relative to other priming agents.

**Figure 4:**
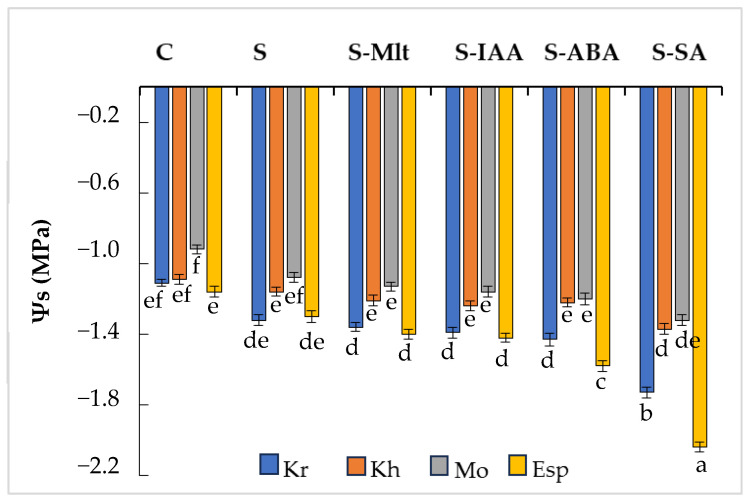
Osmotic potential (Ψs) in durum wheat cultivars (Kr: Karim, Kh: Khiar, Mo: Mocho, Esp: Espelta) grown under control conditions (C), subjected to drought stress (S), or exposed to drought stress following seed priming with melatonin (S-Mlt), indole-3-acetic acid (S-IAA), abscisic acid (S-ABA), or salicylic acid (S-SA). Different lowercase letters above the bars indicate statistically significant differences at *p* < 0.05 according to Fisher’s Least Significant Difference (LSD) test. Error bars represent the standard error of the mean (*n* = 10 plants).

Osmotic potential (Ψs) also decreased under drought stress in all cultivars, by 19%, 6%, 17%, and 12% in Kr, Kh, Mo, and Esp, respectively, compared to control plants (Figure 4). In Kr, this reduction reached 23% in S-Mlt, 25% in S-IAA, 29% in S-ABA, and 56% in S-SA plants. In Kh, Ψs decreased by 11% in S-Mlt, 14% in S-IAA, 12% in S-ABA, and 26% in S-SA plants. In Mo, reductions were 23% in S-Mlt, 26% in S-IAA, 30% in S-ABA, and 43% in S-SA plants. In Esp, decreases were more pronounced, with 21% in S-Mlt, 22% in S-IAA, 36% in S-ABA, and 76% in S-SA plants (Figure 4).

To better discriminate among the studied cultivars and identify the most effective priming agent, osmotic adjustment capacity (OAC) was calculated as an indicator of treatment-induced osmotic adjustment under drought stress. Table 3 shows that all hormonal treatments significantly enhanced OAC under drought conditions compared with non-primed drought-stressed plants (*p* < 0.05). Relative to stressed plants (S), melatonin (Mlt) increased OAC by 1.2 to 1.7 times, indole-3-acetic acid (IAA) by 1.3 to 2.1 times, and abscisic acid (ABA) by 1.5 to 3.0 times in Esp. Salicylic acid (SA) showed the strongest effect, increasing OAC by 2.5 to 6.3 times in Esp (Table 3). Overall, SA was the most effective priming agent across all cultivars, while Esp exhibited the strongest response to hormonal priming treatments.

### 2.3. Oxidative Stress and Antioxidant Activity

Hydrogen peroxide (H_2_O_2_) levels in the shoots of durum wheat increased significantly under drought stress in all cultivars (+45% in Kr, +48% in Kh, +39% in Mo, and +47% in Esp compared to control plants). Seed hormonal priming significantly reduced H_2_O_2_ accumulation, although the extent of reduction varied among cultivars and treatments. In Kr, H_2_O_2_ levels remained only slightly above control values (+11% in S-Mlt and S-IAA, +9% in S-ABA, and +5% in S-SA). In Mo, increases were limited to +12% (S-Mlt), +19% (S-IAA), +18% (S-ABA), and +14% (S-SA) relative to controls. In Kh, the mitigation effect was less pronounced, with H_2_O_2_ levels of +19% (S-Mlt), +35% (S-IAA), +20% (S-ABA), and +33% (S-SA) compared to control plants.

**Figure 5:**
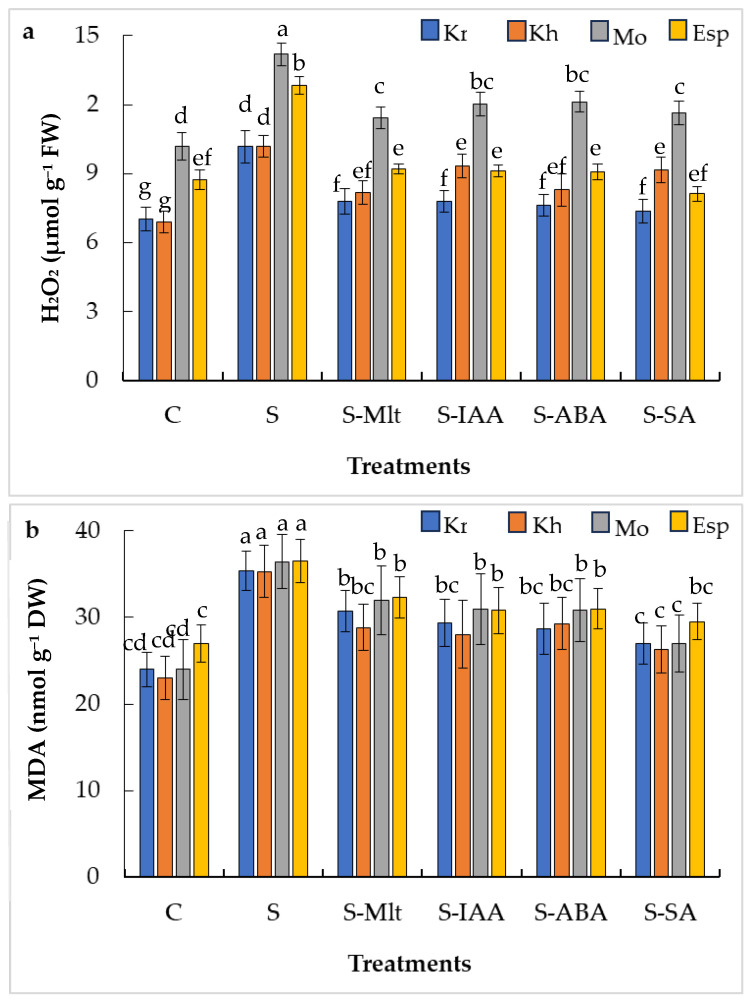
Hydrogen peroxide (H_2_O_2_; (**a**)) and malondialdehyde (MDA; (**b**)) accumulation in durum wheat cultivars (Kr: Karim, Kh: Khiar, Mo: Mocho, Esp: Espelta) grown under control conditions (C), subjected to drought stress (S), or exposed to drought stress following seed priming with melatonin (S-Mlt), indole-3-acetic acid (S-IAA), abscisic acid (S-ABA), or salicylic acid (S-SA). Different lowercase letters above the bars indicate statistically significant differences at *p* < 0.05 according to Fisher’s Least Significant Difference (LSD) test. Error bars represent the standard error of the mean (*n* = 6 plants).

Overall, salicylic acid (SA) was the most effective priming agent, bringing H_2_O_2_ levels closest to those of control plants, particularly in Kr (Figure 5a).

Malondialdehyde (MDA) is a key indicator of cell membrane damage associated with oxidative stress. As shown in Figure 5b, drought stress significantly increased MDA accumulation in all durum wheat cultivars (*p* < 0.05), with a comparatively smaller effect in Esp (+48% in Kr, +54% in Kh, +94% in Mo, and +35% in Esp relative to control plants).

Seed hormonal priming markedly reduced MDA accumulation compared with drought-stressed plants. With melatonin (Mlt), MDA levels decreased to +28% in Kr, +25% in Kh, +33% in Mo, and +20% in Esp (relative to controls). Indole-3-acetic acid (IAA) further reduced MDA accumulation to +22% in Kr and Kh, +29% in Mo, and +14% in Esp. Similarly, abscisic acid (ABA) lowered MDA levels to +20% in Kr, +27% in Kh, +29% in Mo, and +15% in Esp. Salicylic acid (SA) was the most effective priming agent, reducing MDA accumulation to +13% in Kr, +14% in Kh, +13% in Mo, and +9% in Esp relative to control plants (Figure 5b).

**Figure 6:**
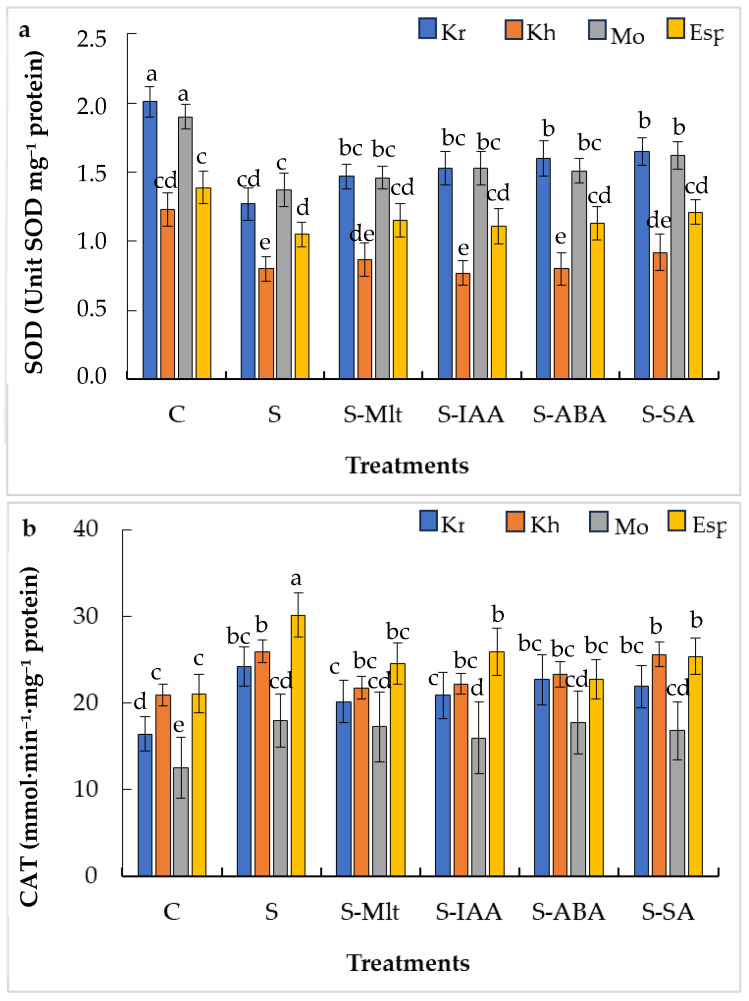
Superoxide dismutase (SOD; (**a**)) and catalase (CAT; (**b**)) activities in durum wheat cultivars (Kr: Karim, Kh: Khiar, Mo: Mocho, Esp: Espelta) grown under control conditions (C), subjected to drought stress (S), or exposed to drought stress following seed priming with melatonin (S-Mlt), indole-3-acetic acid (S-IAA), abscisic acid (S-ABA), or salicylic acid (S-SA). Different lowercase letters above the bars indicate statistically significant differences at *p* < 0.05 according to Fisher’s Least Significant Difference (LSD) test. Error bars represent the standard error of the mean (*n* = 6 plants).

Regarding antioxidant enzymes, Figure 6a shows that drought stress significantly reduced superoxide dismutase (SOD) activity in all cultivars, with varying magnitudes (*p* < 0.05). Esp was the least affected, exhibiting a 24% decrease in SOD activity (relative to control plants), followed by Mo (−28%), Kh (−35%), and Kr (−37%).

Seed hormonal priming mitigated the deleterious effects of drought stress on SOD activity, with responses depending on the priming agent. Melatonin (Mlt) resulted in a recovery of 5–10% of the SOD activity lost under drought conditions. Indole-3-acetic acid (IAA) improved SOD activity by up to 13% in Kr, while abscisic acid (ABA) enhanced recovery by up to 16% in the same cultivar. Salicylic acid (SA) showed the highest efficiency, restoring 10–19% of SOD activity under drought stress (Figure 6a).

Figure 6b illustrates catalase (CAT) activity. A significant stimulation of CAT activity was observed in all cultivars subjected to drought stress (*p* < 0.05), with increases ranging from 24% in Kh to 43% in Mo and Esp, and reaching 47% in Kr. Seed priming slightly reduced CAT activity in drought-stressed plants compared to non-primed stressed plants; however, CAT activity remained higher than in control plants. Regardless of treatment, the cultivar Esp exhibited the highest CAT activity, particularly under drought stress without seed priming.

**Figure 7:**
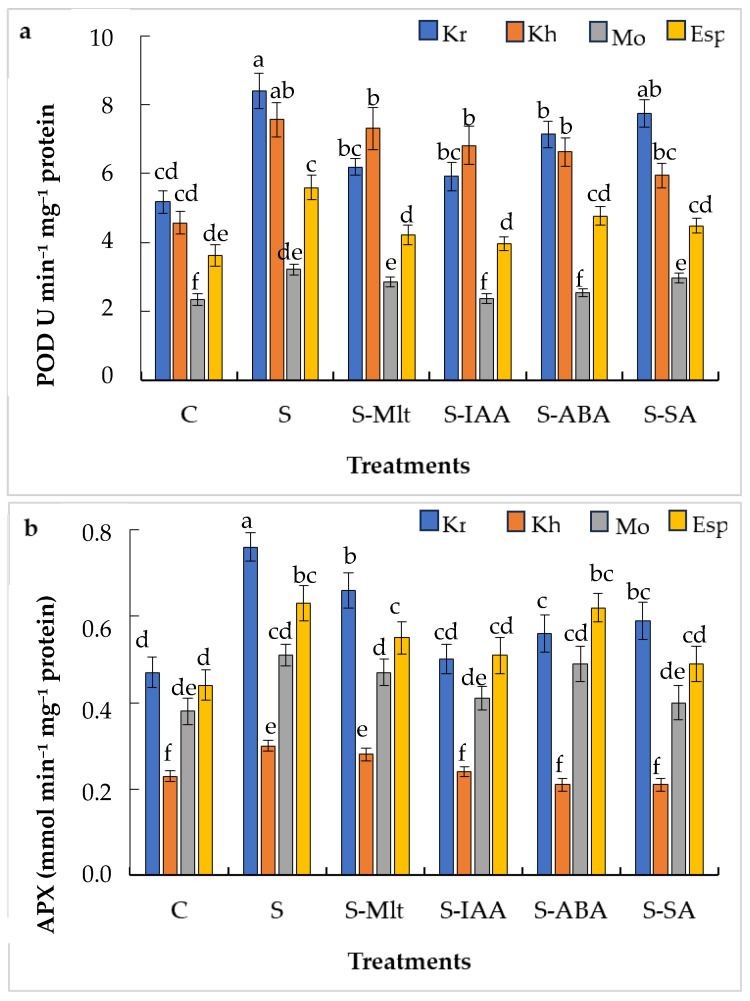
Peroxidase (POD, (**a**)) and ascorbate peroxidase (APX, (**b**)) activities in durum wheat cultivars (Kr: Karim, Kh: Khiar, Mo: Mocho, Esp: Espelta), subjected (S), or not (C) to drought stress, or subjected to drought stress after priming seeds with melatonin (S-Mlt), indole-3-acetic acid (S-IAA), abscisic acid (S-ABA), or salicylic acid (S-SA). Different lowercase letters among bars represent statistically significant differences (*p* < 0.05) according to Fisher’s Least Significant Difference Test. The standard error of the mean of 6 plants is represented by the bars on the columns.

Peroxidase (POD) activity displayed a trend similar to that of CAT. This enzyme activity increased significantly under drought stress (*p* < 0.05) in all cultivars (+ 62% in Kr, +65% in Kh, +37% in Mo, and +54% in Esp compared to control plants; Figure 7a). Regardless of the priming agent, POD activity remained elevated under drought stress compared to control conditions, although to a lesser extent than in non-primed stressed plants.

Under drought stress, melatonin (Mlt) increased POD activity by 20%, 60%, 22%, and 17% in Kr, Kh, Mo, and Esp, respectively (compared to control plants). Indole-3-acetic acid (IAA) enhanced POD activity by 14%, 49%, 1%, and 9%, respectively. Abscisic acid (ABA) increased POD activity by 38%, 45%, 8%, and 32%, respectively, while salicylic acid (SA) led to increases of 50%, 30%, 26%, and 24% in Kr, Kh, Mo, and Esp, respectively (all compared to control plants).

Ascorbate peroxidase (APX) represents another isoform of peroxidase activity. Under drought stress, durum wheat cultivars exhibited a significant increase in APX activity (*p* < 0.05; Figure 7b). Compared to control plants, APX activity increased by 62%, 30%, 34%, and 43% in Kr, Kh, Mo, and Esp, respectively.

Under both control and drought stress conditions, the cultivars Kr and Esp displayed higher APX activity than Kh and Mo. Seed priming with hormonal agents maintained APX activity at levels higher than those of control plants, but lower than those observed in non-primed stressed plants, depending on the priming agent used (Figure 7b).

Among the tested treatments, melatonin appeared to be the most effective, enhancing APX activity by 40%, 22%, 24%, and 25% in Kr, Kh, Mo, and Esp, respectively, under drought stress compared to control conditions.

### 2.4. Osmolytes Accumulation

Glycine betaine (GB) content increased under drought stress. This increase was not significant in Kr (+8%, *p* > 0.05), but was significant (*p* < 0.05) in Kh, Mo, and Esp (+19%, +16%, and +10%, respectively) compared to control plants. However, hormonal seed priming reduced GB levels to values close to, or even lower than, those of control plants across all cultivars and priming agents (Table 4).

Proline, the second osmolyte analyzed in this study, exhibited a pattern similar to that of GB. Under drought stress, proline content increased significantly (*p* < 0.05) by approximately 29%, 17%, 36%, and 20% in Kr, Kh, Mo, and Esp, respectively, compared to control plants (Table 4). A significant reduction in proline concentration was observed in S-Mlt, S-IAA, and S-ABA treatments, with levels falling below those of control plants, whereas in S-SA-treated plants, proline levels remained comparable to controls (Table 4).

Starch and soluble sugars represent two interconvertible forms of carbon. Under drought stress, durum wheat plants showed a significant increase (*p* < 0.05) in shoot starch content (+34% in Kr, +36% in Kh, +18% in Mo, and +14% in Esp compared to control plants; Table 4). Seed priming with Mlt, IAA, and SA restored starch levels to values slightly above those of control plants. In contrast, ABA treatment maintained starch at elevated levels, exceeding even those observed in drought-stressed plants (Table 4).

In contrast to starch, soluble sugars showed an opposite trend under drought stress, with a significant decrease (−15%, −19%, −18%, and −12% in Kr, Kh, Mo, and Esp, respectively; *p* < 0.05) compared to control plants (Table 4). Hormonal seed priming enhanced soluble sugar accumulation, with increases ranging from 11% to 25% in S-Mlt, 3% to 25% in S-IAA, 4% to 28% in S-ABA, and 15% to 34% in S-SA treatments (Table 4). Notably, the highest increases were consistently recorded in Esp, regardless of the priming agent, with the greatest effect observed under SA treatment.

**Figure 8:**
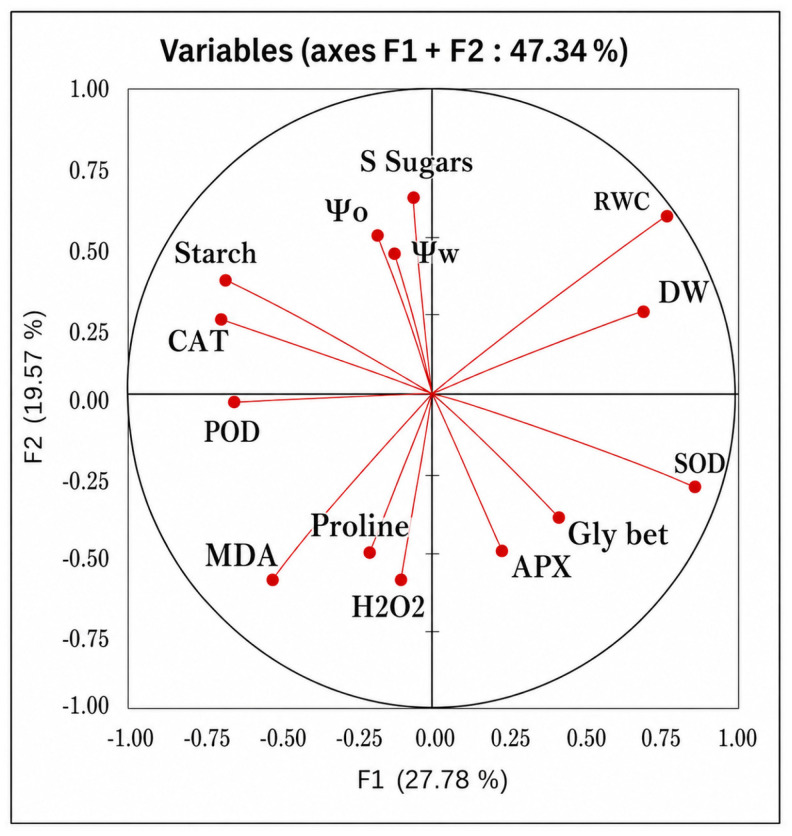
Correlation circle of the first two principal components illustrating relationships among physiological, biochemical, and metabolic traits in durum wheat under drought stress. The first two components (F1 = 27.78% and F2 = 19.57%) together explain 47.35% of the total variance. Vectors oriented in the same direction indicate positive correlations between variables, whereas opposite directions indicate negative correlations. Nearly orthogonal vectors suggest weak or no correlation.

To improve understanding of the mechanisms underlying tolerance and sensitivity of durum wheat to water stress, and to elucidate the physiological and biochemical relationships among the measured parameters, a global principal component analysis (PCA) was performed. Figure 8 presents the correlation circle.

The first two principal components explained 47.34% of the total variance (F1 = 27.78%, F2 = 19.57%). Indeed, the PCA is used primarily as an exploratory and visualization tool to illustrate relationships among variables and to highlight general patterns of treatment separation. Relative water content (RWC), dry weight (DW), and superoxide dismutase (SOD) loaded strongly and positively on F1, reflecting a gradient associated with growth performance and favorable water status. In contrast, malondialdehyde (MDA) and hydrogen peroxide (H_2_O_2_), together with proline and ascorbate peroxidase (APX), were loaded negatively on F1, indicating oxidative stress and cellular damage.

Along F2, soluble sugars (S sugars), water potential (Ψw), and osmotic potential (Ψs) were positively associated, suggesting osmotic adjustment and solute accumulation. Conversely, MDA, H_2_O_2_, and proline were positioned in the negative region of F2, further reflecting stress-induced damage.

**Figure 9:**
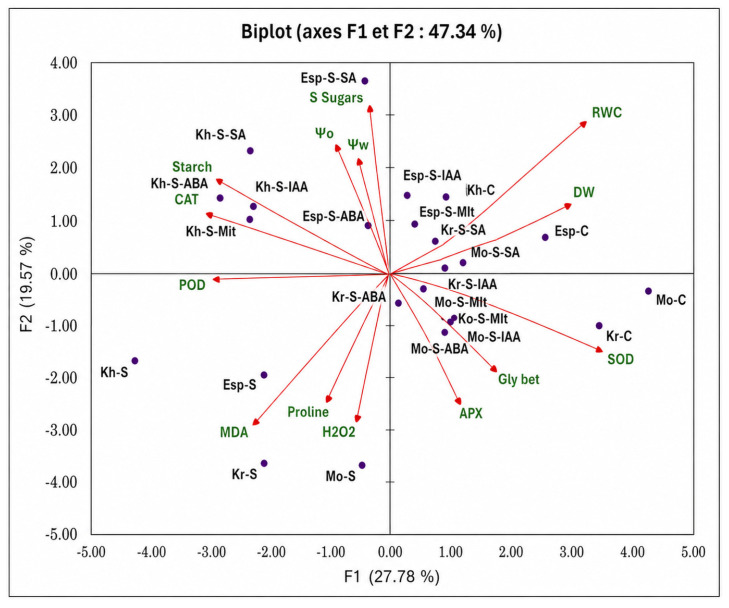
Biplot of the first two principal components illustrating relationships among physiological, biochemical, and metabolic traits, cultivars, and treatments in durum wheat under drought stress. The first two components (F1 = 27.78% and F2 = 19.57%) together explain 47.35% of the total variance. Vectors pointing in the same direction indicate positive correlations between variables, whereas opposite directions indicate negative correlations. Nearly orthogonal vectors suggest weak or no correlation. The red arrows indicate the measured traits, while the black dots represent the cultivars under their respective treatments.

The biplot presented in Figure 9 shows a clear separation between treatments, with control samples positioned on the right and drought-stressed samples on the left. Exogenous application of hormonal priming agents (Mlt, IAA, ABA, and SA) shifts the stressed samples toward the control cluster, primarily by improving water-related physiological traits. Figure 9 highlights a distinct separation between stressed and non-stressed plants along the first principal component (F1), driven by the opposite contributions of growth- and water-status-related traits versus oxidative stress markers. The second component (F2) further distinguishes contrasting adaptive responses, reflecting a trade-off between osmotic adjustment mechanisms and indicators of cellular damage.

**Figure 10:**
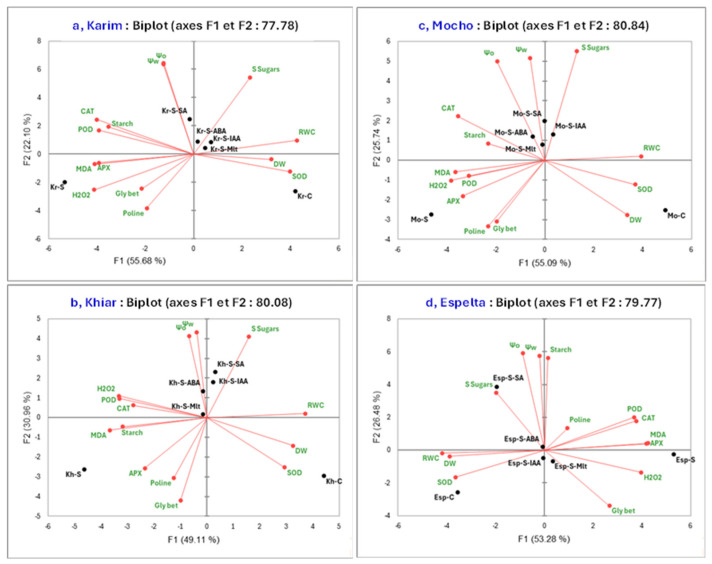
Biplots of the first two principal components illustrating relationships among physiological, biochemical, and metabolic traits, cultivars, and treatments in durum wheat subjected to drought stress. Vectors pointing in the same direction indicate positive correlations between variables, whereas opposite directions indicate negative correlations. Nearly orthogonal vectors suggest weak or no correlation. The red arrows indicate the measured traits, while the black dots represent the cultivars under their respective treatments.

To further elucidate the mechanisms underlying durum wheat responses to drought stress, with a particular focus on cultivar-specific differences, a separate PCA was performed for each genotype. In this analysis, the first two principal components (F1 and F2) accounted for a larger proportion of the total variability than in the global analysis (approximately 77–81%, depending on genotype: Karim 77.8%, Mocho 80.8%, Khiar 80.1%, and Espelta 79.8%; Figure 10). For all cultivars, the control treatment (C) was positioned on the positive side of F1, at the periphery of the ordination space, associated with dry weight (DW), soluble sugars, relative water content (RWC), and superoxide dismutase (SOD). In contrast, drought-stressed plants (S) were located on the opposite side (negative F1), also at the periphery, and were associated with oxidative stress markers, antioxidant responses, and osmotic adjustment-related variables. Hormonal priming treatments were generally distributed near the center or along either side of the F1 axis, indicating intermediate physiological states between control and stress conditions.

In the Karim cultivar (Figure 10a), S-ABA, S-IAA, and S-Mlt clustered toward the positive side of F1, closer to the control group and associated variables (sugars, RWC, DW, and SOD), whereas S-SA was positioned further from the center. In Khiar (Figure 10b), S-IAA and S-SA were located on the positive side of F1, aligning with control-associated traits (sugars, RWC, DW, and SOD). In Mocho (Figure 10c), S-Mlt and S-SA were positioned near the center along F1, while S-IAA shifted toward the positive side. Finally, in Espelta (Figure 10d), S-SA was clearly aligned with the control side, approaching the periphery and closely associated with water status parameters (RWC, Ψw, Ψs), growth (DW), soluble sugars, and SOD activity.

## 3. Discussion

### 3.1. Integrated Responses of Durum Wheat to Drought Stress: Physiological and Biochemical Aspects

In the context of climate change, the impact of drought on plant growth and yield has become increasingly significant, and exploring new approaches to improve crop resilience has also garnered particular attention. Drought stress is a major limiting factor for durum wheat (*T. turgidum*) productivity by reducing water availability, restricting stomatal conductance, and impairing carbon assimilation, ultimately leading to reductions in growth, biomass, and photosynthetic activity [41]. Present results do not escape this general behavior, with demonstrated significant reduction in plant growth, spad index, chlorophyll pigments, shoot hydration, and increased H_2_O_2_ production and MDA accumulation in all durum wheat cultivars subjected to drought stress, with some differences. The expression of beneficial metabolic functions that support plant growth (SPAD, chlorophyll, RWC, Ψs, Ψw, etc.) decreased, against an increase in the expression of destructive functions (H_2_O_2_, MDA, etc.). Antioxidant enzymes are stimulated to increase for some and to decrease for others. Under these conditions of water stress, and without any intervention, genotypic variation was evident in our study, where Espelta maintained the highest dry biomass, followed by Karim, Mocho, and Khiar. These differential responses are consistent with genetic diversity observed in wheat and other cereals, which influence water-use efficiency, osmotic adjustment, and antioxidant activation [42]. Accordingly, Spanic et al. [43] showed that water deficit leads to chloroplast destabilization, decreased photosynthetic pigments, and reduced leaf metabolic capacity under drought stress. Water deficit leads to a decline in relative water content, chlorophyll pigment concentration, osmotic potential, and water potential, and it also reduces leaf transpiration rate [19]. Otherwise, drought disrupts nutrient mineralization, diffusion, and flow in the soil, thereby limiting their uptake by plants [44,45]. It also decreases cell membrane permeability, transpiration flux, and active transport processes, resulting in reduced nutrient mobilization within plant tissues [46]. Under water-limited conditions, chlorophyll content decreased by 13–15% due to the activation of chlorophyllase and the inactivation of key enzymes [47]. The decreasing gradient from Esp to Kh in beneficial traits (SPAD, chlorophyll, RWC, Ψs, Ψw, sugars, proline, CAT, POD, etc.) mirrored increases in destructive indicators (H_2_O_2_, MDA), indicating that stronger drought tolerance is associated with better water status and lower oxidative damage. A key physiological consequence of drought is the disruption of cellular redox homeostasis, resulting in excess production of reactive oxygen species (ROS) such as hydrogen peroxide (H_2_O_2_). This ROS accumulation can cause extensive membrane lipid peroxidation, as evidenced by increased malondialdehyde (MDA) levels under water deficit [48,49,50]. The excessive electrons are transferred to oxygen, generating ROS that react with proteins, lipids, membranes, and nucleic acids. These oxidative reactions damage chloroplasts, mitochondria, and other organelles, alter membranes, inactivate metabolic enzymes, and may ultimately trigger programmed cell death [51,52,53] and suppress chlorophyll biosynthesis [54,55]. However, ROS and MDA, widely recognized signs of membrane damage [56,57], are commonly used markers of oxidative stress in wheat, and recent investigations have confirmed that drought-induced oxidative damage correlates with impaired metabolic processes and compromised growth traits [58,59]. Indeed, cultivars with lower ROS and MDA levels under stress presumably possess more robust antioxidant defenses, which mitigate oxidative damage. Condition met by the cultivar Esp (and Kr in a second line) in the present study, and supported by the preserved water relations, growth, and photosynthetic traits under drought. Several authors reported that osmolyte accumulation helps maintain cellular turgor, protects macromolecular structures, and supports metabolic functions under environmental stress [60,61].

### 3.2. Contribution of Hormonal Priming to the Adaptation of Durum Wheat to Drought Conditions

The genotypic differences observed in this study, which present Esp and Kr cultivars as the most resilient and Kh as sensitive to drought stress, were maintained when seeds were primed with hormonal agents. At this level, all used hormones promoted plant growth, water relations, and antioxidant traits, and clearly mitigated the oxidative stress (Less H_2_O_2_ and MDA accumulation). However, their beneficial actions differ and remain dependent on the physiological and biochemical traits.

The protective effects of SA are attributed to its function as a signaling molecule that modulates plant stress responses, enhancing tolerance via osmoprotectant synthesis (e.g., proline, soluble sugars) and upregulation of antioxidant enzymes that detoxify ROS [62,63]. Specifically, SA has been shown to increase osmolyte content and antioxidant capacity while reducing H_2_O_2_ and MDA accumulation, thereby limiting oxidative damage under drought stress [64,65]. Accordingly, the genotype-dependent responses to SA priming observed in this study reflect the influence of genetic background on hormone sensitivity and stress adaptation pathways [66]. Current results highlighted the beneficial effects of SA through enhanced osmotic adjustment and antioxidant enzyme activities (APX, POD, CAT, SOD), thereby mitigating oxidative stress damage. The observed increases in APX, POD, CAT, and SOD activities align with reports that SA enhances enzyme activity to maintain redox balance under drought stress [67,68]. These enzymes collectively scavenge superoxide radicals and H_2_O_2_, reducing oxidative injury and preserving membrane integrity.

Exogenous ABA application as a seed priming agent maintained higher leaf relative water content (RWC) under drought conditions compared to non-primed plants. This improvement is associated with enhanced osmotic adjustment (OA). Accordingly, recent studies confirmed that drought priming enhances cell water retention by improving osmotic adjustment through increased proline and glycine betaine biosynthesis pathways [66]. Similarly, ABA-treated *Camellia oleifera* seedlings showed improved proline and soluble sugar accumulation and better osmotic balance under drought stress while reducing lipid peroxidation markers [69]. These findings support the role of ABA in metabolic reprogramming that strengthens drought resilience through osmolyte accumulation. Otherwise, ABA priming enhanced antioxidant enzyme activities, including superoxide dismutase (SOD), catalase (CAT), ascorbate peroxidase (APX), and peroxidase (POD), thereby reducing oxidative stress markers. Similar findings were reported in sugarcane seedlings, where ABA treatment enhanced antioxidant gene expression and improved ROS scavenging capacity under drought stress [70]. ABA-treated Camellia plants also showed enhanced SOD and POD activities and reduced membrane damage [69]. Recent transcriptomic analyses reveal that ABA priming induces alternative splicing events and activates ABA-responsive gene networks, facilitating faster stress responses during subsequent drought exposure [71]. These molecular adjustments suggest the establishment of stress memory mechanisms that enhance plant adaptation to recurring drought conditions. Thus, it is suggested that ABA priming enhances drought tolerance through coordinated osmotic adjustment, osmolyte accumulation, ROS regulation, and antioxidant enzyme activation. The overall used cultivars responded positively to ABA seed priming with some genotypic differences. This can explain the superiority of Esp when considering biomass production, where S-ABA plants increased shoot DW by 15%, as compared to S plants, whereas no more than 6% registered in Kr plants (+3% in Kh and +0.8% in Mo S-ABA plants, as compared to S plants).

Several studies provide direct evidence of IAA-mediated drought tolerance. For example, *Pseudomonas aeruginosa* strains producing IAA significantly improved drought tolerance in *Vigna radiata*, with enhanced photosynthetic activity, antioxidant efficiency, and plant growth metrics under water-limited conditions [72]. Gene-level studies also reinforce the role of IAA: overexpression of the TrIAA27 gene in *Arabidopsis thaliana* increased biomass, drought tolerance, and stress-responsive signaling pathways [73]. Experimental work in wheat further demonstrates that exogenous IAA restores relative water content, fresh weight, and affects antioxidant enzyme activities, showing cultivar-specific drought tolerance responses [74]. Without escaping these responsive mechanisms, we suggest that IAA priming enhances drought tolerance by regulating osmolyte accumulation, improving osmotic adjustment, stabilizing chlorophyll and membranes, sustaining plant growth, limiting reactive oxygen species (ROS) accumulation, and activating the antioxidative stress system.

For melatonin, this substance is endowed with multifunctional roles that span direct antioxidative protection, physiological regulation, hormonal interaction, membrane stabilization, and genomic reprogramming, making it an effective agent for improving plant resilience under water-limited conditions. In line with the present study, Muhammad et al. [75] reported in maize seedlings that melatonin priming significantly increased SOD, POD, CAT, glutathione, and ascorbate activities while reducing ROS, lipid peroxidation, and electrolyte leakage, improving growth and chlorophyll stability under drought. Reviews confirm activation of MAPK signaling and drought-responsive transcription factors [76]. Melatonin enhances osmotic adjustment, photosynthesis, and membrane stability. In foxtail millet, melatonin increased chlorophyll content, photosynthetic rate, and biomass [77]. Studies in multiple crops highlight enhanced photosynthesis, antioxidant activity, and growth traits under drought through hormonal interaction [78]. Otherwise, Mlt has demonstrated active transcription factors (HSFA1s, DREBs), modulated proteomic elements (HSPs, glycolytic enzymes), and adjusted ion homeostasis genes (NHX1, AKT1), improving germination vigor and stress tolerance [79]. In triticale, melatonin priming (20 µM) increased germination rate, radicle and plumule growth, photosynthetic rate, and antioxidant activity while reducing ROS and MDA under drought stress [80]. Indeed, Melatonin reinforces antioxidant defenses, supports osmotic regulation, enhances photosynthetic performance, mediates hormonal interaction, and reprograms stress signaling, making it an effective drought-priming agent.

**Figure 11:**
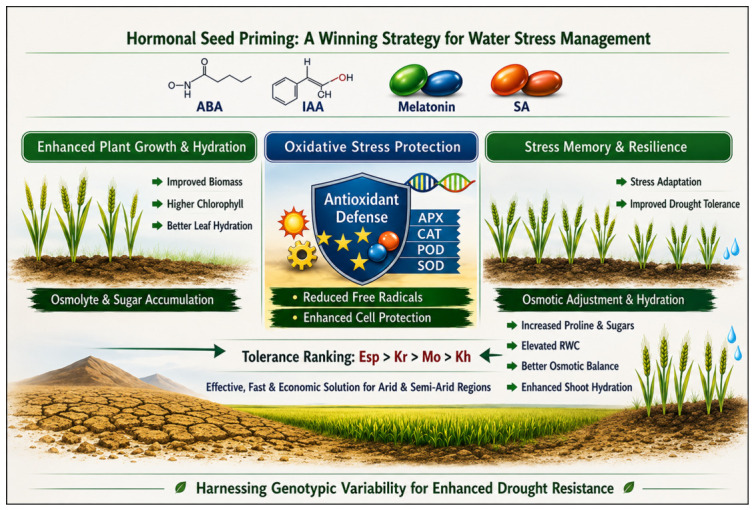
Mechanisms of hormonal seed priming in durum wheat response to drought stress (Figure generated by Copilot: https://copilot.microsoft.com/, consulted on 20 March 2026).

Overall, the results support the conclusion that drought tolerance in durum wheat is strongly associated with the maintenance of water status, photosynthetic pigments, and antioxidative capacity. ABA, IAA, Mlt, and SA completely meet these conditions by conferring enhanced resilience through modulating biochemical pathways that buffer against water deficit and oxidative stress. We suggest that combining genetic selection with targeted priming treatments, especially hormones like ABA, IAA, Mlt, and SA, can significantly enhance drought tolerance in durum wheat, through improving osmotic balance, preserving photosynthetic machinery, and fortifying antioxidant defenses. This approach offers practical avenues to sustain wheat performance in arid and semi-arid agrosystems, where water scarcity represents the major problem. The variation observed among cultivars in this study indicates the involvement of distinct drought tolerance mechanisms, consistent with previous reports highlighting significant genotype-dependent differences in traits such as relative water content (RWC), osmotic adjustment, and chlorophyll content. Cultivars that maintain higher RWC and osmotic potential under water deficit generally exhibit improved growth and yield, owing to more efficient water retention and the preservation of cellular homeostasis [81,82]. This metabolic balance relies on effective osmotic adjustment to sustain cellular hydration, along with a strong antioxidant capacity that protects cellular structures and ensures the proper functioning of essential metabolic processes, including photosynthesis. In this context, all hormonal priming treatments applied in the present study proved effective in enhancing these physiological responses, although with slight variations in their magnitude of impact. Among the cultivars, Esp and, to a lesser extent, Kr showed a greater inherent tolerance to soil water deficit and displayed a more pronounced responsiveness to the priming treatments. Figure 11 illustrates the modes of action of phytohormones used as priming agents, as well as the underlying mechanisms responsible for the observed differences among cultivars.

## 4. Materials and Methods

### 4.1. Plant Material

Plant material and seed priming and experimental design: Durum wheat (*Triticum turgidum* ssp. *durum* Desf.) was used as plant material for this experiment. Four cultivars (two Tunisian, Karim and Khiar, and two Spanish, Mocho and Espelta) were used to evaluate their response to drought stress and highlight the extent of hormonal seed priming in these conditions: The seeds (supplied by the National Institute for Field crops, Boussalem, Tunisia) were incubated for 6 h, at room temperature, in Petri dishes containing filter paper soaked in the coresponding solution (ABA: 1.5 mM, IAA: 1.5 mM, Mlt: 0.1 mM, SA: 1.5 mM), and then dried again for 48 h at room temperature. The seeds allocated to the control (C) or stressed (S) treatments were also incubated in deionized water. The experiment was conducted in a greenhouse in the Centro de Edafología y Biología Aplicada del Segura (CEBAS-CSIC, Murcia, Spain), with a mean temperature of 28–31 °C/18–21 °C day/ night and a natural photoperiod of 13/11 h. Hormone concentrations were previously optimized in the Lab [83,84]. The seeds allocated to the control (C) or stressed (S) treatments were also incubated in deionized water. The pots were placed in a system equipped with automated irrigation and subjected to different treatments within a three-factorial design experiment (water levels, priming agent, and cultivars), arranged in a completely randomized design with three replications per treatment (three pots of 20 plants each). Six treatments were in total applied to all cultivars: control plants (C), stressed plants (S), stressed plants with ABA-primed seeds (S-ABA), stressed plants with IAA-primed seeds (S-IAA), stressed plants with Mlt-primed seeds (S-Mlt), and stressed plants with SA-primed seeds (S-SA). Measurements were conducted with ten replicates for most parameters, whereas oxidative and antioxidant traits were assessed using six replicates.

During the first seven weeks, all plants were irrigated at the field capacity (FC), then drought stress was applied by decreasing the irrigation to 25% field capacity (FC) for two weeks.

### 4.2. Field Capacity Determination

Field capacity (FC) is defined as the amount of water retained in soil after excess gravitational water has drained away (typically 24–72 h after saturation). It is a key parameter in irrigation and plant physiology studies. FC was determined using the gravimetric method according to Klute [85]. Air-dried soil was first sieved (2 mm) and placed into plastic pots equipped with drainage holes at the bottom. The soil was then gradually saturated with distilled water until complete saturation was achieved, ensuring minimal disturbance of soil structure. Following saturation, the pots were left to drain freely under laboratory conditions for 48 h, allowing excess gravitational water to percolate out of the soil profile. After the drainage period, the moist soil was weighed to obtain the fresh weight (*W*_1_). Subsequently, soil samples were oven-dried at 105 °C for 24 h to a constant weight and reweighed to determine the dry weight (*W*_2_). Field capacity was expressed as gravimetric water content using the following equation:

$$\mathrm{FC}\;(\%)=\frac{W_{1}-W_{2}}{W_{2}}\times 100$$

To induce drought stress, the amount of irrigation water was reduced to 25% FC.

### 4.3. Biomass Production and Water Relations

For growth quantification, samples of fresh shoots were weighed and then dried at 70 °C for 72 h to estimate dry weight biomass.

Leaf relative water content was assessed following the procedure of Jones and Turner [86]. A single leaf was excised from the mid-section of each plant and immediately placed in plastic bags to prevent moisture loss. The fresh weight (FW) was recorded directly after sampling. Leaves were then immersed in distilled water in Petri dishes and incubated in darkness for 24 h to achieve full turgidity. After this period, the turgid weight (TW) was measured. The samples were subsequently oven-dried at 70 °C until a constant weight was reached, and the dry weight (DW) was determined.

The relative water content was calculated using the formula:

$$RWC=\frac{\left(FW-DW\right)}{\left(TW-DW\right)}\times \;100$$

Leaf water potential (ψw) was determined following the method of Scholander et al. [87]. Measurements were taken on the upper, fully expanded leaves, approximately two hours after sunrise, using a pressure chamber (Model C52-SF, WESCOR Inc., South Logan, UT, USA). For osmotic potential (Ψs), leaf samples were harvested in the field, immediately placed in ice-cooled containers, and transported to the laboratory under chilled conditions. The leaf samples were homogenized (ground) in acetone, then centrifuged at 4000 g for 20 min at 4 °C. The osmotic pressure of the resulting supernatant was determined using an osmometer. For each measurement, 10 µL of the sample was diluted with 90 µL of Milli-Q water to obtain a final volume of 100 µL. The values obtained were subsequently converted to osmotic potential (MPa) using the Van’t Hoff equation.

Osmotic adjustment capacity was calculated by subtracting Ψs of control plants from that of treated plants (S, S-ABA, S-IAA, S-Mlt, or S-SA).

### 4.4. SPAD Index

The SPAD index (Soil–Plant Analysis Development) is a non-destructive measurement of relative chlorophyll content based on leaf greenness intensity and using a handheld device, SPAD-502 (Konica Minolta, Tokyo, Japan). Ten plants per treatment were used to calculate the mean.

### 4.5. Photosynthetic Pigments Analysis

Photosynthetic pigments were quantified following the procedure described by Dere et al. [88]. Briefly, 50 mg of fresh leaf tissue were homogenized in 10 mL of 80% acetone and centrifuged at 2500 rpm for 10 min. The resulting supernatant was collected, and absorbance (A) was recorded at three wavelengths (470, 645, and 662 nm) using 80% acetone as a blank. Pigment concentrations were subsequently calculated using the following equations.

Chl a (µg/gFW) = (11.75 × A663) − (2.35 × A647)

Chl b (µg/gFW) = (18.61 × A645) − (3.96 × A662)

Car (µg/gFW) = (1000 × A470) − (2.27 × chl a) − (81.4 × chl b)/227

### 4.6. Determination of Starch and Soluble Sugar

Starch content was quantified by enzymatic hydrolysis followed by colorimetric detection according to Haissig and Dickson [89]. Dried pellets were resuspended in 760 µL double-distilled water and 100 µL MES (0.5 M, pH 5.0), then supplemented with 40 µL amyloglucosidase. Suspensions were incubated with agitation at 30 °C for 16 h. 100 µL of the supernatant was diluted with 200 µL water, and released glucose was determined using the Anthrone assay: 500 µL of freshly prepared 0.2% Anthrone in 97% H_2_SO_4_ was added under a fume hood, mixtures were cooled on ice for 1 min, vortexed, and 200 µL was transferred to a microplate for absorbance reading at 625 nm. The concentrations were calculated from a standard curve (0.025–0.125 mg/mL) and expressed as starch equivalents relative to sample mass.

Soluble sugars were quantified following the Anthrone colorimetric method described by Hodge and Hofreiter [90]. A volume of 100 µL of the liquid-phase supernatant was transferred into standard microcentrifuge tubes. The standard curve was prepared using glucose concentrations ranging from 0.1 to 1.00 mg/mL by mixing appropriate volumes of a 1 µg/µL glucose solution with ethanol. Each tube received 500 µL of freshly prepared 0.2% Anthrone reagent in 97% sulfuric acid. This step was performed under a fume hood due to the highly exothermic nature of the reaction. Samples were immediately cooled on ice for 1 minute. After vortexing, 200 µL of each reaction mixture was transferred to a microplate, and absorbance was measured at 625 nm within two hours of reagent addition. The concentration of soluble sugars was calculated from the standard curve and expressed relative to the sample’s fresh weight.

### 4.7. Determination of Glycine Betaine Analysis

Glycine betaine content was determined following a modified protocol of Grattan [91]. Lyophilized plant samples (20 mg dry weight) were rehydrated with 1 mL of double-distilled water and incubated at 30 °C under agitation for 24 h. After centrifugation at 4600 rpm for 5 min, 100 µL of the supernatant was transferred to labeled microtubes and mixed with 400 µL of 1 N sulfuric acid. A standard curve was prepared using betaine at 1 µg/µL by dissolving 10 mg of betaine in 10 mL of bidistilled water. To each 100 µL aliquot of the standard, 400 µL of 1N sulfuric acid (H_2_SO_4_) is added. Samples and standards were kept on ice for 1 h, then treated with 250 µL of freshly prepared KI-I_2_ solution. Tubes were incubated at 4 °C for 16 h to allow crystal formation, followed by centrifugation at 10,000 rpm for 15 min at 0 °C. The supernatant was discarded, and the crystals were dissolved in 2 mL of 1,2-dichloroethane. After agitation, 200 µL of the organic phase was transferred to quartz microplate wells, and absorbance was measured at 365 nm using a Biotek spectrophotometer (Winooski, Vermont, USA).

### 4.8. H_2_O_2_ and MDA

Lyophilized tissue (50 mg) was homogenized in 1 mL of 0.1% trichloroacetic acid (TCA), vortexed twice for 10 s, and centrifuged at 12,000 rpm for 15 min at 4 °C. The supernatant was used for H_2_O_2_ and MDA determination.

Hydrogen peroxide (H_2_O_2_) levels were quantified as an indicator of oxidative damage following a modified iodometric assay. For each sample, 100 µL of potassium phosphate buffer (50 mM, pH 7.0), 500 µL of potassium iodide (1 M), 100 µL of diluted extract (1:1 with 0.1% TCA), and 300 µL of Milli-Q water were combined, mixed thoroughly, and incubated in the dark for 1 h. After incubation, 200 µL of the reaction mixture was transferred to microplate wells, and absorbance was measured at 390 nm. A standard curve was prepared using serial dilutions of H_2_O_2_ in 0.1% TCA. Concentrations were calculated from the standard curve and expressed as nmol H_2_O_2_ per gram dry weight.

Malondialdehyde (MDA) was quantified following Hodges et al. [92] using the thiobarbituric acid (TBA) assay with TCA correction. Two aliquots of 125 µL supernatant were dispensed into labeled heat-resistant microtubes: one received 125 µL of 20% trichloroacetic acid (TCA), and the other 125 µL of a TCA/TBA mixture (20% TCA containing 0.5% TBA). Tubes were incubated in a 90 °C water bath for 1 h, then cooled on ice. From each reaction, 180 µL was transferred to microplate wells, and absorbance was recorded at 440, 532, and 600 nm. MDA concentration was calculated by subtracting the TCA-only blank from the TCA/TBA reaction to correct for non-MDA chromogens, applying the specific absorbance coefficients, and expressing results as nmol MDA per gram fresh weight.

### 4.9. Determination of Antioxidant Enzymes

#### 4.9.1. Enzyme Extraction

According to the method adapted from Torres et al. [93], approximately 500 mg of leaf tissue was ground in liquid nitrogen and homogenized in 5 mL of 50 mM MES/KOH buffer (pH 6.0) containing 40 mM KCl, 2 mM CaCl_2_, and 1 mM L-ascorbic acid. After centrifugation at 13,000× *g* for 10 min at 4 °C, the supernatant was stored at −80 °C until use.

#### 4.9.2. Superoxide Dismutase (SOD) Activity

Total superoxide dismutase (SOD) activity was assayed according to Scebba et al. [94]. The reaction was initiated by combining 20 µL of enzymatic extract with 10 µL of 100 mM HEPES buffer (pH 7.8), 20 µL of 5 mM NTB, 80 µL of 10 mM xanthine, 68.4 µL of distilled water, and 1.6 µL of xanthine oxidase, to obtain a final volume of 200 µL.

The experimental design included three control reactions to ensure accurate determination of superoxide dismutase (SOD) activity. Blank 1 contained all reagents except the enzymatic extract, which was replaced with MES buffer. Blank 2 included all reagents except xanthine oxidase, which was substituted with MES buffer. Blank 3 omitted both the enzymatic extract and xanthine oxidase, with MES buffer added instead. A reagent mixture (excluding both extract and enzyme) was prepared, and 180 µL was dispensed into each well. The reaction was initiated by adding xanthine oxidase, followed by thorough mixing. Absorbance was measured at 560 nm immediately before and 10 min after enzyme addition. SOD activity was then calculated and expressed as units of SOD per mg of protein.

#### 4.9.3. Ascorbate Peroxidase (APX) Activity

Ascorbate peroxidase (APX) activity was assayed as described by Miyake and Asada [95], in a final reaction volume of 200 µL containing 60 µL of 50 mM potassium phosphate buffer (pH 7.0), 40 µL of 5 mM L-ascorbic acid, 30 µL of 1 mM EDTA, and 50 µL of hydrogen peroxide (0.34%). The reaction was initiated by adding 20 µL of the enzymatic extract. For blank controls, the extract was replaced with MES buffer. A reaction mixture (excluding the extract) was prepared as a master cocktail, and 180 µL was dispensed into each well prior to initiating the reaction. APX activity was determined by monitoring the decrease in absorbance at 290 nm, corresponding to the oxidation of L-ascorbic acid. Enzyme activity was calculated and expressed as mmol min^−1^ mg^−1^ protein.

#### 4.9.4. Catalase (CAT) Activity

Catalase (CAT) was assayed as described by Chance and Maehly [96], using the same enzymatic extract in a final reaction volume of 200 µL, consisting of 110 µL of 50 mM potassium phosphate buffer (pH 7.0), 70 µL of hydrogen peroxide, and 20 µL of enzymatic extract to initiate the reaction. A reagent cocktail containing the buffer and H_2_O_2_ was prepared, and 180 µL was dispensed into each well prior to enzyme addition. For blank controls, the enzymatic extract was replaced with MES buffer. CAT activity was determined by monitoring the decrease in absorbance at 240 nm, corresponding to the decomposition of H_2_O_2_. Enzyme activity was calculated and expressed as mmol min^−1^ mg^−1^ protein.

#### 4.9.5. Peroxidase (POD) Activity

Peroxidase (POD) activity was determined using a continuous spectrophotometric method adapted from Frick [97], with guaiacol and hydrogen peroxide as substrates. The assay measures the rate of guaiacol oxidation catalyzed by POD in the presence of H_2_O_2_, monitored at 480 nm, where the oxidized product exhibits a brown coloration. The molar extinction coefficient of tetraguaiacol was taken as 5.57 × 10^3^ M^−1^ cm^−1^.

The reaction mixture was prepared by supplementing 0.05 M sodium phosphate buffer (pH 7.0) with 300 µL of 33% (*v*/*v*) hydrogen peroxide and 250 µL of guaiacol. For each assay, 195 µL of this reaction mixture was dispensed into a microplate well, and the reaction was initiated by adding 5 µL of enzymatic extract. Absorbance was recorded at 480 nm at 1 min intervals for 5 min. POD activity was calculated and expressed as units of enzyme activity (POD U min^−1^ mg^−1^ protein) per mg of protein.

### 4.10. Artificial Intelligence Use

The design of the graphical conclusion was made with the help of Copilot, with some modifications.

### 4.11. Statistical Analysis

Data and statistical analyses were performed using the software XLSTAT (2025.1). All data are presented as mean ± standard error. Analysis of variance (ANOVA) was performed to check whether the effects of the treatment (C, S, S-ABA, S-IAA, S-Mlt, S-SA) on the respective factors were significant. The significance of differences among treatments was determined by Fisher’s least significant difference test (LSD) at 5%. Means were declared significantly different when the difference between any two treatments was more important than the LSD value generated from the ANOVA.

## 5. Conclusions

The present study demonstrates that hormonal seed priming can enhance drought resilience in durum wheat, thereby supporting the initial hypothesis. Despite cultivar-dependent variability and trait-specific responses, priming treatments consistently improved key physiological parameters, including plant growth, chlorophyll content, and plant water status under water deficit conditions. The concurrent reduction in drought-induced oxidative stress provides further evidence that these treatments contribute to improved stress tolerance through enhanced antioxidant defense mechanisms.

Priming with ABA, IAA, melatonin, and salicylic acid was associated with improved leaf hydration, maintenance of photosynthetic efficiency, and increased antioxidant capacity, which together contributed to more stable metabolic functioning under drought. These findings are consistent with the concept that seed priming may induce a form of physiological “stress memory,” enabling plants to respond more effectively to subsequent water deficit, although this mechanism requires further validation at the molecular level.

The magnitude of the priming effect varied among cultivars, highlighting the importance of cultivar × treatment interactions. Among the tested cultivars, the Spanish Esp exhibited the highest drought tolerance and responsiveness to priming, followed by the Tunisian cultivar Kr, while Kh appeared to be the most sensitive. Accordingly, cultivars can be ranked in decreasing order of drought tolerance as: Esp > Kr > Mo > Kh.

While these results confirm the beneficial effects of hormonal seed priming under controlled, short-term drought conditions, further studies are needed to elucidate the underlying biochemical and molecular mechanisms, particularly those linking early priming events to later developmental responses. In addition, evaluation under field conditions, including yield performance and post-stress recovery, is essential to assess the agronomic relevance and stability of these effects across environments and growth stages.

## Figures and Tables

**Table 1:** Concentrations of chlorophyll a (Chl-a), chlorophyll b (Chl-b), and carotenoids in durum wheat cultivars (Kr: Karim, Kh: Khiar, Mo: Mocho, Esp: Espelta) grown under control conditions (C), subjected to drought stress (S), or exposed to drought stress following seed priming with melatonin (S-Mlt), indole-3-acetic acid (S-IAA), abscisic acid (S-ABA), or salicylic acid (S-SA). Different letters within each column indicate statistically significant differences at *p* < 0.05 (*n* = 10).

	Chl a (mg g^−1^ FW)				Chl b (mg g^−1^ FW)				Car (mg g^−1^ FW)			
	Kr	Kh	Mo	Es	Kr	Kh	Mo	Es	Kr	Kh	Mo	Es
C	3.73 ± 0.31 ^a^	4.27 ± 0.27 ^c^	4.27 ± 0.33 ^a^	3.97 ± 0.31 ^b^	1.73 ± 0.11 ^c^	2.00 ± 0.10 ^d^	2.11 ± 0.18 ^a^	1.80 ± 0.11 ^d^	0.63 ± 0.052 ^a^	1.19 ± 0.11 ^b^	0.95 ± 0.077 b	1.03 ± 0.068 ^b^
S	3.30 ± 0.27 ^c^	3.99 ± 0.23 ^d^	3.6 ± 0.22 ^e^	3.65 ± 0.17 ^c^	1.66 ± 0.13 ^cd^	1.99 ± 0.12 ^d^	1.56 ± 0.12 ^c^	1.91 ± 0.13 ^c^	0.49 ± 0.038 ^d^	0.90 ± 0.066 ^d^	0.70 ± 0.054 c	0.82 ± 0.068 ^d^
S-Mlt	3.60 ± 0.25a ^ab^	5.08 ± 0.37 ^ab^	3.61 ± 0.26 ^c^	4.51 ± 0.33 ^a^	1.78 ± 0.11 ^c^	2.34 ± 0.18 ^b^	2.03 ± 0.14 ^a^	2.27 ± 0.18 ^a^	0.58 ± 0.031 ^b^	1.42 ± 0.11 ^a^	1.13 ± 0.11 ^a^	1.15 ± 0.12 ^a^
S-IAA	3.89 ± 0.31 ^a^	5.44 ± 0.41 ^a^	3.06 ± 0.21 ^d^	3.98 ± 0.28 ^b^	1.93 ± 0.15 ^b^	2.62 ± 0.19 ^a^	1.85 ± 0.13 ^b^	1.90 ± 0.11 ^c^	0.68 ± 0.041 ^a^	1.48 ± 0.012 ^a^	0.58 ± 0.043 ^d^	0.90 ± 0.076 ^c^
S-ABA	3.51 ± 0.22 ^b^	5.01 ± 0.42 ^b^	4.03 ± 0.21 ^b^	3.73 ± 0.25 ^bc^	2.20 ± 0.18 ^a^	2.25 ± 0.19 ^bc^	1.92 ± 0.11 ^b^	2.08 ± 0.16 ^b^	0.48 ± 0.033 ^d^	1.05 ± 0.088 ^c^	1.12 ± 0.088 ^a^	1.07 ± 0.061 ^b^
S-SA	3.52 ± 0.24 ^b^	4.81 ± 0.39 ^b^	3.62 ± 0.30 ^c^	3.87 ± 0.23 ^b^	1.58 ± 0.11 ^d^	2.14 ± 0.17 ^c^	1.79 ± 0.13 ^b^	2.05 ± 0.14 ^b^	0.53 ± 0.035 ^c^	0.99 ± 0.066 ^c^	0.85 ± 0.066 ^b^	1.02 ± 0.085 ^b^

**Table 2:** Relative water content (RWC) in shoots of durum wheat cultivars (Kr: Karim, Kh: Khiar, Mo: Mocho, Esp: Espelta) grown under control conditions (C), subjected to drought stress (S), or exposed to drought stress following seed priming with melatonin (S-Mlt), indole-3-acetic acid (S-IAA), abscisic acid (S-ABA), or salicylic acid (S-SA). Different letters within each column indicate statistically significant differences at *p* < 0.05 (*n* = 10).

Treatments		Cultivars		
	Kr	Kh	Mo	Esp
C	94 ± 6.62 ^a^	91 ± 7.33 ^a^	93 ± 6.72 ^a^	96 ± 7.2 ^a^
S	63 ± 5.34 ^c^	57 ± 4.55 ^c^	65 ± 5.23 ^c^	69 ± 5.63 ^c^
S-Mlt	80 ± 6.72 ^b^	74 ± 5.53 ^b^	79 ± 5.77 ^b^	87 ± 6.17 ^b^
S-IAA	82 ± 7.24 ^b^	73 ± 5.91 ^b^	78 ± 5.83 ^b^	85 ± 7.12 ^b^
S-ABA	80 ± 6.81 ^b^	75 ± 6.17 ^b^	77 ± 5.77 ^b^	82 ± 6.51 ^b^
S-SA	86 ± 7.15 ^ab^	79 ± 5.82 ^b^	81 ± 6.32 ^b^	91 ± 7.27 ^ab^

**Table 3:** Osmotic adjustment capacity (OAC), calculated as the difference in osmotic potential (Ψs) between control and drought-stressed plants, in durum wheat cultivars (Kr: Karim, Kh: Khiar, Mo: Mocho, Esp: Espelta) grown under control conditions (C), subjected to drought stress (S), or exposed to drought stress following seed priming with melatonin (S-Mlt), indole-3-acetic acid (S-IAA), abscisic acid (S-ABA), or salicylic acid (S-SA). Different letters within each column indicate statistically significant differences at *p* < 0.05 (*n* = 10).

Treatments		Cultivars		
	Kr	Kh	Mo	Esp
S	0.21 ± 0.017 ^d^	0.07 ± 0.005 ^c^	0.16 ± 0.011 ^d^	0.14 ± 0.011 ^d^
S-Mlt	0.25 ± 0.015 ^c^	0.12 ± 0.011 ^b^	0.21 ± 0.016 ^cd^	0.24 ± 0.021 ^c^
S-IAA	0.28 ± 0.021 ^bc^	0.15 ± 0.012 ^b^	0.24 ± 0.020 ^c^	0.26 ± 0.022 ^c^
S-ABA	0.32 ± 0.024 ^b^	0.13 ± 0.009 ^b^	0.28 ± 0.023 ^b^	0.42 ± 0.027 ^b^
S-SA	0.62 ± 0.037 ^a^	0.28 ± 0.021 ^a^	0.40 ± 0.031 ^a^	0.88 ± 0.066 ^a^

**Table 4:** Accumulation of glycine betaine, proline, starch, and soluble sugars in shoots of durum wheat cultivars (Kr: Karim, Kh: Khiar, Mo: Mocho, Esp: Espelta) grown under control conditions (C), subjected to drought stress (S), or exposed to drought stress following seed priming with melatonin (S-Mlt), indole-3-acetic acid (S-IAA), abscisic acid (S-ABA), or salicylic acid (S-SA). Different letters within each column indicate statistically significant differences among treatments (*n* = 10; *p* < 0.05).

**Treatments**	**Glycine Betaine (mg g^−1^ DW)**				**Starch (mg g^−1^ DW)**			
	**Kr**	**Kh**	**Mo**	**Es**	**Kr**	**Kh**	**Mo**	**Es**
C	98 ± 6.2 ^a^	89 ± 5.8 ^b^	126 ± 8.8 ^c^	123 ± 9.4 ^b^	11.9 ± 0.81 ^c^	17.8 ± 1.13 ^c^	15.1 ± 1.22 ^b^	17.1 ± 1.25 ^b^
S	106 ± 7.3 ^a^	105 ± 6.9 ^a^	146 ± 10 ^a^	136 ± 11.1 ^a^	15.9 ± 1.12 ^a^	24.2 ± 1.53 ^a^	17.8 ± 1.31 ^a^	19.5 ± 1.53 ^a^
S-Mlt	88 ± 6.5 ^b^	56 ± 3.7 ^c^	108 ± 9.3 ^d^	137 ± 11 ^a^	13.5 ± 0.92 ^b^	20.6 ± 1.24 ^bc^	16.1 ± 1.11 ^b^	17.9 ± 1.21 ^b^
S-IAA	94 ± 7.4 ^ab^	47 ± 3.3 ^d^	123 ± 8.9 ^c^	138 ± 10.3 ^a^	13.0 ± 0.88 ^b^	18.0 ± 1.22 ^c^	15.9 ± 1.14 ^b^	17.6 ± 1.54 ^b^
S+-ABA	89 ± 6.3 ^b^	58 ± 4.1 ^c^	135 ± 6 ^b^	129 ± 8.7 ^b^	12.9 ± 0.76 ^b^	20.8 ± 1.55 ^bc^	15.4 ± 1.12 ^b^	17.9 ± 1.33 ^b^
S-SA	101 ± 7.7 ^a^	61 ± 4.9 ^c^	122 ± 9.4 ^c^	113 ± 9.2 ^c^	16.0 ± 1.21 ^a^	21.9 ± 1.46 ^b^	18.7 ± 1.23 ^a^	20.0 ± 1.67 ^a^
**Treatments**	**Proline (mg g^−1^ DW)**				**Soluble Sugars (mg g^−1^ DW)**			
	**Kr**	**Kh**	**Mo**	**Es**	**Kr**	**Kh**	**Mo**	**Es**
C	612.5 ± 32 ^b^	482.2 ± 35.4 ^b^	376.3 ± 21.7 ^c^	334.4 ± 29.4 ^b^	58.4 ± 5.2 ^b^	94.6 ± 6.6 ^c^	95.6 ± 5.7 ^ab^	93.1 ± 6.4 ^b^
S	789.3 ± 41.2 ^a^	565.0 ± 41.4 ^a^	512.2 ± 38.9 ^a^	401.0 ± 31.5 ^a^	49.8 ± 3.7 ^c^	76.6 ± 5.8 ^d^	78.7 ± 5.5 ^b^	81.8 ± 5.9 ^c^
S-Mlt	388.4 ± 27.6 ^c^	383.5 ± 27.3 ^c^	299.9 ± 20.3 ^d^	199.6 ± 12.4 ^c^	65.0 ± 4.1 ^a^	105.6 ± 8.4 ^bc^	108.7 ± 8.3 ^a^	116.4 ± 8.4 ^ab^
S-IAA	248.8 ± 18.5 ^e^	345.7 ± 23.7 ^d^	360.9 ± 27.8 ^c^	190.9 ± 15.2 ^c^	59.9 ± 4.9 ^b^	110.2 ± 8.8 ^b^	113.3 ± 9.2 ^a^	116.1 ± 7.6 ^ab^
S+-ABA	351.9 ± 22.6 ^d^	396.8 ± 30.1 ^c^	363.5 ± 26.5 ^c^	339.0 ± 27.6 ^b^	60.8 ± 3.9 ^b^	102.8 ± 6.9 ^c^	112.9 ± 9.5 ^a^	119.4 ± 9.0 ^a^
S-SA	582.2 ± 33.8 ^b^	480.9 ± 36.4 ^b^	401.0 ± 21.2 ^b^	348.9 ± 27.7 ^b^	67.2 ± 5.3 ^a^	122.0 ± 8.7 ^a^	118.3 ± 8.7 ^a^	125.1 ± 10.3 ^a^

## Data Availability

The original contributions presented in this study are included in the article. Further inquiries can be directed to the corresponding author.

## Abbreviations

The following abbreviations are used in this manuscript:

ABA	Abscisic acid
APX	Ascorbate peroxidase
C	Control
CAT	Catalase
Esp	Espelta
GB	Glycine betaine
IAA	indole-32-acetic acid
Kh	Khiar
Kr	karim
Mlt	Melatonin
Mo	Mocho
POD	Peroxidase
OA	Osmotic adjustment
OAC	Osmotic adjustment capacity
ROS	Reactive oxygen species
S	Stressed plants
SA	salicylic acid
S-ABA	Stressed plants with ABA-primed seeds
S-IAA	Stressed plants with IAA-primed seeds
S-Mlt	Stressed plants with Mlt-primed seeds
S-SA	Stressed plants with SA-primed seeds
